# Medical students' examinations during the pandemic: Performance and perceptions of anatomy teaching and learning before, during, and after COVID‐19 lockdowns

**DOI:** 10.1002/ase.70136

**Published:** 2025-10-16

**Authors:** Michał Ciucias, Katarzyna Polak‐Boroń, Marcelina Łopińska, Jerzy Stanisław Gielecki, Anna Żurada

**Affiliations:** ^1^ Department of Anatomy and Histology Collegium Medicum, Faculty of Medicine, Warmia and Mazury University Olsztyn Poland; ^2^ Individual Specialist Medical Practice Olsztyn Poland

**Keywords:** anatomical sciences, anatomy teaching, COVID‐19, learning performance, medical education

## Abstract

Numerous methods of distance learning were introduced during the COVID‐19 pandemic that critically impacted the medical education landscape. This study assessed the anatomy learning performance of first‐year medical students in both theoretical and practical examinations before, during, and after COVID‐19 lockdowns and surveyed their perceptions and experiences of online anatomy courses. Participants comprised 725 first‐year medical students, with 435 from the Polish Division (PD) and 290 from the English Division (ED), and were initially organized into four cohorts according to the anatomy course completion year: 2019, 2020, 2021, and 2022. Looking into educators' and students' teaching and learning experiences during the pandemic, a major performance reduction was observed among students learning online, which lasted throughout the academic year following the resumption of in‐person classes. The practical aspects of online examinations using digital images showed the most pronounced reduction in performance, whereas the change in theoretical examination scores was less marked. The shift to online learning exposed underlying curriculum shortcomings that persisted even after the return to in‐person classes, highlighting the need for comprehensive curricular revisions to sustain improvements in anatomy education. This study contributes to the ongoing discussion on constantly improving the efficacy and quality of teaching and learning anatomy in the ever‐changing medical education landscape.

## INTRODUCTION

The SARS‐CoV‐2 or coronavirus disease 2019 (COVID‐19) pandemic started unexpectedly, affecting entire education systems worldwide. Prior to COVID‐19, no methods had been established to cope with the type of operating conditions experienced during the pandemic outbreak. When the pandemic lockdowns started, the anatomy curricula were promptly reorganized to match the online teaching and learning environment. The curricular modifications require careful planning and implementation to maintain educational effectiveness, and their effect on academic outcomes, especially examination performance, is less well understood.^1^ In many cases, distance teaching and learning posed challenges because of a lack of prior computer training among teachers and students.^2^

In Poland, shortly after the pandemic outbreak, all in‐person educational activities were withdrawn, remote learning was introduced across all educational institutions, and similar approaches were adopted worldwide.^3^, ^4^ The unprecedented challenge of transitioning teaching methods to virtual environments deprived students and educators of essential learning tools, including dissected and prosected specimens, skeletons, and models,^5^ rendering anatomy teaching and learning challenging.^5^, ^6^ Various alternative modalities were used as replacements for in‐person practical activities, including digitized, donor‐based resources,^6^ living anatomy aided by ultrasonography,^7^ video atlases, three‐dimensional (3D) modeling programs, and virtual anatomy dissection tables.^8^ During the pandemic lockdowns, the lack of face‐to‐face interactions between students and teachers was compensated with blended curricula^9^, ^10^ and online learning platforms.^11^, ^12^ Some online solutions comprised live‐streaming of prosections, partially mitigating the lack of hands‐on experience.^13^

In response to lockdown‐induced disruptions, the Department of Anatomy at the University of Warmia and Mazury in Poland adopted a comprehensive approach that integrated Moodle‐based interactive videos, quizzes, and synchronized proctoring with Microsoft Teams and Safe Exam Browser.^14^ Although these tools were broadly used before the pandemic, their synergistic use, combined with locally produced instructional videos, staff and student tutorials, and spotter images, represented an integrated approach to maintaining the anatomy curriculum.

The pandemic‐related challenges affected the teaching and learning process, which, in turn, had implications for assessment. This raised new concerns about the delivery of examinations and student performance in the online setting.

Before the pandemic, medical students' knowledge was successfully examined using computers and other electronic devices, such as multimedia tablets, in both remote and in‐person environments.^15^ Some studies have reported that student performance was better in computer‐based examinations than in traditional paper‐based examinations,^16^, ^17^ in contrast to other studies where no differences between the traditional and digitized methods of student assessment were observed.^18^, ^19^

Online theoretical examinations are easier to conduct than practical ones, but maintaining proper standards can be challenging because of poor face‐to‐face interaction and the absence of personal supervision. This increases the risk of unauthorized examination content sharing and other forms of academic dishonesty.^20^ Cheating in online examinations can be detected with the help of eye‐tracking software, and the examinee's computer can be restricted to display only the examination interface.^21^ Other methods to mitigate the possibility of cheating include randomizing the order of questions and presenting them individually^20^ and imposing time limits for each question and the entire examination.^22^

Conducting the practical examinations in an online environment requires much greater commitment from the teaching staff. Digital replacement for actual specimens must be provided. This was accomplished with digital images^6^; however, the need to access an adequate volume and quality^18^ of photographs of anatomical specimens can be a major obstacle in conducting practical examinations in the online environment. The inability to interact with the specimens during practical examinations makes it difficult to properly recognize the marked structure and its topographical relationships, though this could be mitigated by using 3D digital applications, as previously demonstrated.^23^

Despite multiple studies describing teaching and learning methods adopted during the pandemic^6^, ^24^, ^25^, ^26^ and addressing student perceptions of these adaptations,^27^, ^28^, ^29^ few have directly compared the scores obtained in anatomy summative assessments before, during, and after COVID‐19 lockdowns. Recently, Chaker et al.^30^ compared student performance on summative assessments in lockdowns‐imposed online‐only cohorts and in subsequent post‐lockdowns blended classroom cohorts with pre‐pandemic, in‐person cohorts; the authors provided evidence that the blended classroom cohort outperformed both the online and in‐person cohorts. García et al.^31^ examined summative assessment scores in a first‐year medical Cell Biology course in pre‐ and post‐lockdowns cohorts, observing decreased scores in the latter. Changes in the examination scores between pre‐ and during‐pandemic periods have also been addressed previously, but with no reference to the post‐pandemic period.^23^, ^32^ As a result, there remains a gap in evaluating student examination performance in anatomy after the post‐lockdowns return to in‐person classes, particularly when compared to online learning during the lockdowns and to pre‐lockdown in‐person classes.

### Relevance

The shift to online teaching and learning during the outbreak of COVID‐19 not only accelerated the implementation of digital technologies but also exposed the need to revisit existing teaching methods and evaluate their impact on current students' learning and examination performance.^30^, ^33^ By capturing both the experiences and outcomes of students who studied online during lockdowns and those who enrolled after the return to in‐person classes, this study offers an opportunity for post‐pandemic curriculum refinement. Identifying how the design of the teaching and assessment methods affects student examination performance can effectively guide educators in addressing any potential curriculum issues rather than merely reverting to pre‐pandemic routines. Although the need for the development of a robust anatomy curriculum has already been acknowledged,^34^ the innovations that emerged during COVID‐19 must continue to be adapted into the current anatomical pedagogy.^35^ Consequently, the results of this study underscore the necessity of reshaping anatomy curricula to fit into the post‐pandemic educational landscape, and the need for robust curricular adaptations has been identified previously.^10^, ^36^, ^37^ Current results contribute to the ongoing discourse on how the teaching and learning practices introduced during the pandemic can effectively continue in current educational settings.^38^

### Study objectives

This study aimed to review the adaptation and delivery of a first‐year medical anatomy course under COVID‐19 conditions with three objectives. The primary objective was to compare first‐year medical students' examination scores and passing rates during online learning with the performance in the first year of enrollment after returning to in‐person learning. Secondary objectives encompassed exploring student‐reported responses to online learning mode, including their learning resources, examination preferences, study time, and pandemic‐related challenges, and analyzing the overall impact of these factors on performance.

### Hypotheses

Given the objectives, the following hypotheses have been formulated:

(Practical—lockdowns vs. pre‐pandemic): During COVID‐19 lockdowns (2020–21), mean practical examination scores and first‐term passing rates are lower than in the pre‐pandemic cohort (2019C).

(Practical—post‐lockdowns): In the first post‐lockdown cohort (2022), practical examination scores and first‐term passing rates remain below pre‐pandemic levels (2019C).

(Theoretical): Theoretical examination performance varies less across cohorts than practical performance.

(Learning resources): Greater use of learning resources (such as departmental instructional videos and textbooks) is associated with a reduced self‐reported risk of failing the final anatomy examination.

(Learning impediments): Higher levels of pandemic‐related impediments are associated with an increased self‐reported risk of failing the final anatomy examination.

Acceptance or rejection of the hypotheses is presented in the Discussion alongside the relevant sections.

## MATERIALS AND METHODS

### Ethical approval

This study was approved by the Scientific Research Ethics Committee of the University of Warmia and Mazury in Olsztyn, Poland (Decision No. 24/2021, November 17, 2021).

### Study context

#### Anatomy course prior to COVID‐19

Medical students at UWM, Olsztyn, Poland, enroll in a first‐year gross anatomy course separated into the Polish Division (PD) and English Division (ED), each admitting about 220 students annually. The PD is a Polish‐language program enrolling domestic students, while the ED is an international, English‐language program admitting students mainly from abroad. Despite differences in language of instruction and student demographics, both divisions are taught by the same faculty and follow the same curriculum. However, PD students are expected to master both Polish and English anatomical terminology, whereas ED students are required to know English terminology only. PD candidates are selected based on high‐school examination results in biology, chemistry, and physics, while ED admits through foreign diplomas or entrance exams in these subjects, with difficulty corresponding to final examinations in Polish high schools.

**FIGURE 1:**
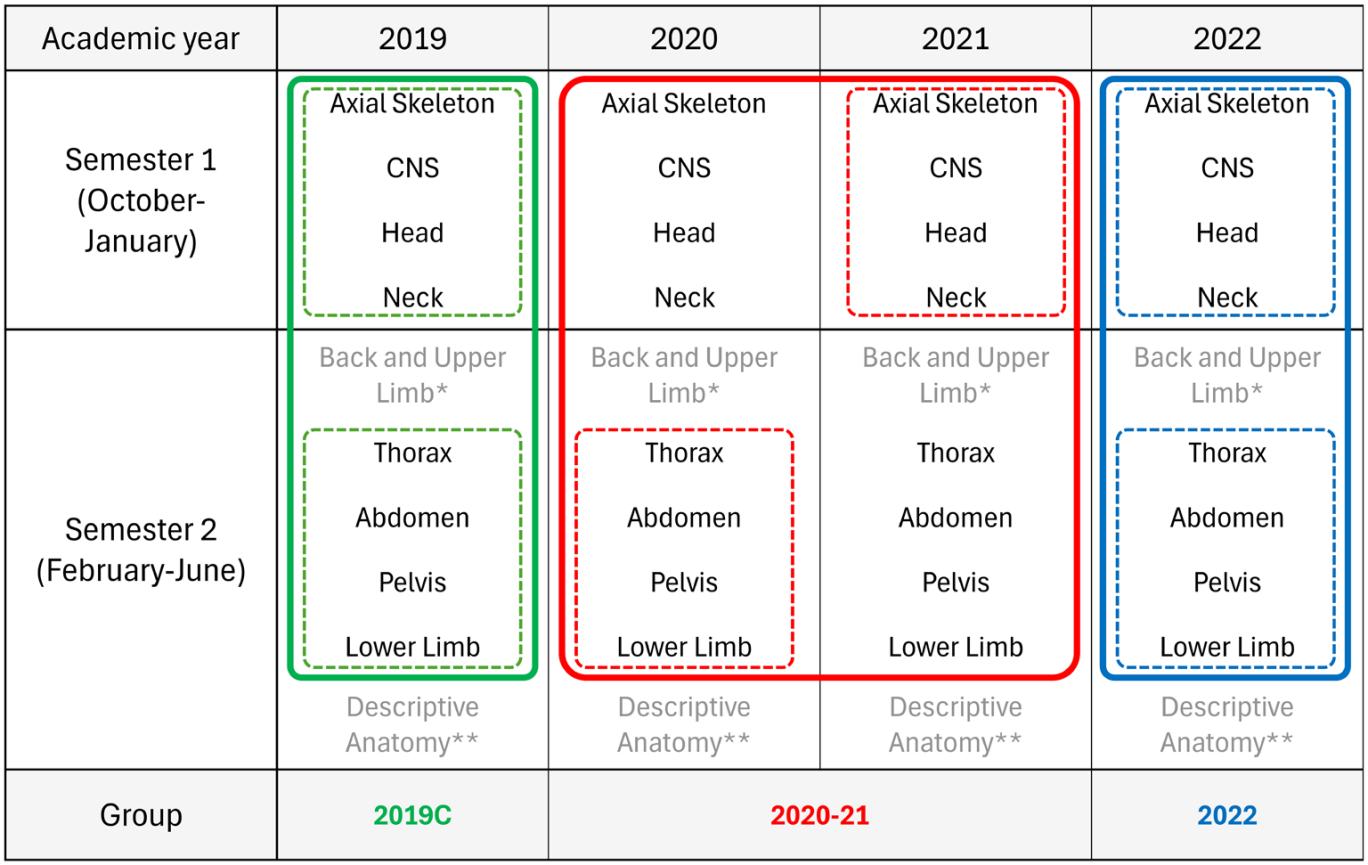
Composition of control and comparison groups. *Has been excluded from analysis, as this unit has not been conducted online. **Has been excluded from analysis because of different scope of anatomy learning when compared to other units. Dashed lines mark anatomy units that have been chosen for examination score comparisons. Solid lines mark specific academic years and semesters that have been chosen for control (2019C), comparison 1, online (2020–21), and comparison 2, post‐lockdown (2022) groups.

The 30‐week course spans two semesters and includes nine region‐based units, as well as a review unit on system‐based descriptive anatomy (specific units have been described in Figure 1). It involves 160 laboratory and 40 lecture hours, adhering to guidelines from the Faculty Council and the Polish Ministry of Science and Higher Education. The laboratories are organized into bi‐weekly two‐hour sessions in small groups—typically up to five persons—using prosected specimens and radiological images. Prosected specimens used during laboratory sessions were prepared in the departmental dissection room by the anatomy technical staff, using formalin solution embalming. Specimens were rotated every 2–4 years, depending on their state of preservation, and after educational use, donors were buried in accordance with the standards of the departmental body donation program. Accordingly, students in the anatomy course learn from pre‐prepared prosections rather than through a formal dissection course. Dissection opportunities are available only as voluntary activities within the departmental student scientific club, which is open to all interested students.

Laboratory sessions are supplemented by 1.33 h of weekly flipped spotter classes.^39^ A detailed overview of the course curriculum, including laboratory activities, formative and summative assessments, and adaptations across pre‐pandemic, lockdown, and post‐lockdown periods, is provided in Table 1.

**TABLE 1:** Overview of anatomy course and assessment methods before, during, and after COVID‐19 lockdowns.

	Pre‐pandemic	During lockdowns (2020–21)	Post‐lockdowns (2022)
Course delivery	In‐person	Online only	In‐person with some hybrid components
Assessment delivery	In‐person, proctored	Online only, proctored (1:10 proctoring staff ratio)	In‐person, proctored
Lecture hours	40		
Laboratory hours	160		
Lectures schedule	Once a week		
Laboratory schedule	Bi‐weekly two‐hour sessions, supplemented with weekly flipped‐classroom session		
Group size	Up to 10 students		
Teaching staff	One teaching professor per student group. Three groups simultaneously during a laboratory session		
Key resources/tools	Prosected specimens and radiological images; face‐to‐face lectures, lecturer commenting multimedia presentations	Microsoft Teams, Customized Moodle website and Safe Exam Browser; instructional videos; digital specimen images; asynchronous, pre‐recorded lectures	Reintroduced prosected specimens and radiological images; Moodle class‐entrance tests; synchronous, pre‐recorded lectures
Laboratory activities	Small group discussion on prosected specimens and radiological images	Synchronous Microsoft Teams sessions using Moodle with on‐topic videos and interactive specimen image quizzes	Small group discussion on prosected specimens and radiological images
Formative activities	Ungraded, verbal mini‐quizzes issued by teacher with immediate feedback offered	Results of Moodle quizzes and video questions available immediately; multiple attempts possible; feedback from teacher available	Reintroduced pre‐pandemic methods; Moodle content preserved and available; comprehensive feedback sessions; atlas of 3D virtual specimens introduced
Summative assessments	Class‐entrance pen and paper short MCQ test, 10 questions; end‐of‐section examinations with theoretical (20 MTF questions) and practical (16 structures on specimens, 4 on radiology images) components; up to two retakes offered per examination	Structure reflecting that of pre‐pandemic, but delivered with Moodle	Reintroduced pre‐pandemic methods; class‐entrance tests conducted with Moodle
Final examination	Theoretical component, 50 MTF questions; practical component using TOSSPE method, 44 structures on prosected specimens, and six structures on radiological images; up to two retakes offered	Structure reflecting that of pre‐pandemic, but examinations delivered with Moodle	Reintroduced pre‐pandemic methods
Assessment timing	Short test: 10 min; theoretical examination: 30 min; practical examination: 11 min; final examination: 60 and 26 min for theoretical and practical parts, respectively	Time extended by 20% when compared to pre‐pandemic setting	Reintroduced pre‐pandemic methods

Students undergo regular assessments, including short class‐entrance tests and end‐of‐section examinations comprising theoretical (multiple true/false questions (MTF)) and practical components, the latter using the Tablet‐assisted Objective Structured Spotter Practical Examination (TOSSPE) method, which combines a regular spotter examination (including clinical‐based radiology image assessment) with the tablet application, enabling instant and detailed feedback to be given to the students.^40^ End‐of‐section examination questions are noncumulative. The MTF question format was chosen to encourage independent, in‐depth analysis of each statement rather than just recognizing correct answers.^41^, ^42^ Students get a point for correctly answering each statement individually. An example of an MTF question is shown in Table 2. Examination questions were developed by departmental anatomy faculty based on the recommended textbooks and atlases, and (for practical examinations) the anatomical structures studied during laboratory sessions. Each question set was internally peer‐reviewed by other faculty members before administration. Example items from the theoretical and practical examinations are provided in the respective supplementary tables (Tables S1 and S2).

**TABLE 2:** Example of an MTF question.

Question stem	Evaluate the following statements regarding specific nerve damages and their associated symptoms
Statement 1	Damage to the radial nerve typically results in an inability to extend the wrist and fingers
Statement 2	Injury to the axillary nerve often leads to a loss of sensation over the deltoid muscle and difficulty in abducting the arm at the shoulder joint
Statement 3	The ulnar nerve injury is usually associated with “claw hand” deformity and loss of sensation in the thumb
Statement 4	Damage to the vagus nerve commonly results in hyperactive reflexes in the lower limbs
Statement 5	Sciatic nerve damage can cause difficulty in dorsiflexion of the foot

*Note*: In the above example the first, second, and fifth statements are true. The third statement, while partially true (ulnar nerve injury is indeed associated with a “claw hand” symptom), also contains false information: loss of sensation in the thumb is incorrect. The fourth statement is false. This MTF question requires understanding of multiple nerves and their respective symptoms. Assessing this level of understanding with a MCQ question would be challenging, as only knowledge of a single construct (i.e., knowledge of a nerve or symptom) could be assessed at a time.

The passing‐grade threshold is 70%, with two retakes allowed. Since the MTF test has about 50% true and 50% false statements, random guessing would yield an average score of 50%. Setting a threshold at 70% thus creates a clear demarcation between guessing and genuine understanding (in‐department discussion determined that a 60% threshold would still be influenced by possible random guessing).

Near‐peer teaching is preferred, improving academic performance and long‐term knowledge retention for teachers and learners.^43^, ^44^, ^45^

#### Changes in the anatomy course to adapt to COVID‐19

In March 2020, all in‐person activities shifted online due to COVID‐19, shortly after the beginning of the second semester (February to June) of the 2019/2020 academic year. The subsequent first semester (October to January) of the 2020/2021 academic year was also held online, which resulted in first‐year students starting their medical course in the remote format. Finally, the pandemic restrictions were loosened in the second semester of the 2020/2021 year, and anatomy practical classes reverted to the in‐person, dissection room‐based format.

The course schedule, hours, activities, topics, and group organization were maintained during lockdowns. Examination times were extended by 20% to compensate for the apparent cognitive load alteration.

E‐learning forms implemented in the department to maintain teaching, learning, assessment, and communication included instructional videos and digital images delivered through a Moodle (Moodle PTY LTD, Australia) learning‐management system. Microsoft Teams (Microsoft, Redmond, WA, USA) was adopted to facilitate communication between teaching staff and students. During online learning, students preferred textbooks and the department's online lectures for preparing for theoretical examinations. For practical examinations, textbooks remained the prevalent resource, along with instructional videos and ANKI—an open‐source digital flash‐card application that uses a spaced‐repetition algorithm to enhance knowledge retention^46^, ^47^—loaded with labeled images provided by the department, as indicated by participants of the current study.

#### Post‐lockdowns anatomy course

After the lockdowns ended, the course resumed largely to its pre‐pandemic form; however, an attempt was made to reshape the curriculum toward the blended form by retaining the digital modalities introduced during the pandemic. Specifically, instructional videos and image‐based quizzes were reused as supplements to in‐person laboratory activities. Moodle was also used to conduct class‐entrance tests, allowing for prompt review and feedback.

Following the current study's results, the departmental one‐on‐one feedback sessions (between students and teachers) were introduced to support post‐pandemic cohorts in overcoming practical anatomy challenges. Although these were also available pre‐pandemic,^40^ a personalized, in‐person, in‐depth discussion component has been largely extended. This allowed students not only to review their practical exam results directly with teachers and clarify any uncertainties, but also to demonstrate a more profound understanding of the anatomy concepts, which may not have been fully captured during the summative practical examinations. These sessions provide an opportunity for formative assessment and deeper conceptual engagement. Moreover, feedback sessions help identify knowledge gaps and bridge them.

Aside from the feedback sessions, a photogrammetry‐based,^48^ 3D, annotated, virtual specimens collection was established and delivered through Moodle. It is based entirely on departmental resources (cameras, lighting, rotary table, and computers), which allow for the swift creation of virtual specimens. Mirroring departmental specimens and extending the exposure to them beyond class hours, this resource aimed to support spatial understanding and improve alignment between the learning materials and practical assessments.

A detailed comparison of the course curriculum across all three modes has been presented in Table 1.

### Characterization of the study participants

A total of 725 (45.7% male, 54.3% female) first‐year medical students participated in the study: 435 from PD (32.4% male, 67.6% female) and 290 from ED (65.5% male, 34.5% female). While students were given expanded gender options during enrollment, all selected “male” or “female,” which were used for analysis. Students were grouped into four cohorts according to the anatomy course completion year: 2019, 2020, 2021, and 2022 (see Table 3 for details). The mean male‐to‐female ratio in PD was 0.59 (ranging from 0.57 to 0.61), higher than the 0.48 ratio at other Polish medical universities in 2019–2022.^49^ For ED, the ratio was 1.9 (ranging from 1.6 to 2.2), significantly differing from 0.83^49^ in ED students at other Polish medical universities. Polish medical university data encompassed all program years, not exclusively the first year, which might have contributed to this discrepancy in the ED ratio. Both PD and ED students were aged 18–22 years.

**TABLE 3:** Study participant distribution by academic year and gender.

Academic year	Polish Division			English Division		
	*N*	Males	Females	*N*	Males	Females
2019	102	39 (38.24%)	63 (61.76%)	58	40 (68.97%)	18 (31.03%)
2020	95	28 (29.47%)	67 (70.53%)	78	48 (61.54%)	30 (38.46%)
2021	105	31 (29.52%)	74 (70.48%)	94	63 (67.02%)	31 (32.98%)
2022	133	43 (32.33%)	90 (67.67%)	60	39 (65.00%)	21 (35.00%)

### Study design

This study used a retrospective, mixed‐methods approach to investigate how the transition to online learning during COVID‐19 lockdowns affected students' examination scores, passing rates, and attitudes toward this learning mode.

A control group (2019C) and two comparison groups were used in the study. The 2019C group consisted of the 2019 cohort, who completed the course in person before the pandemic. The first comparison group (2020–21) included the 2020 and 2021 cohorts, who completed part of their course online. The second comparison group (2022) consisted of the 2022 cohort, who completed the course in person in the first year enrolled after reverting to in‐person learning. Detailed group compositions are presented in Figure 1.

Scores from theoretical (MTF) and practical (TOSSPE) exams were compared among the groups, focusing on first‐term scores and passing rates.

Admission baseline data were obtained from official statistics provided by the faculty Dean's office. For each cohort and division, the total number of applicants and the number who met admission criteria and enrolled were recorded. Admission decisions in the Polish Division were based on national secondary school final examination results in biology, chemistry, and physics, whereas in the English Division, eligibility was determined by foreign diploma equivalency or a dedicated entrance examination. From these values, the enrolled‐to‐total candidate ratio was calculated and used as a proxy indicator of baseline knowledge level. The cohort with the highest ratio was assigned a baseline weight of 1, and weights for other cohorts were adjusted proportionally (e.g., if 9% = 1, then 7% = 1.3). These weights were then applied when the current study examination scores were compared between groups. Candidate‐level admission scores were not available, and the published minimum entry scores were presented for reference only and were not used in the adjustment (Table 4).

**TABLE 4:** Baseline weight factors used to weight the examination scores.

Academic year	Polish Division				English Division			
	2019	2020	2021	2022	2019	2020	2021	2022
Total candidates	1500	1812	1434	2297	178	173	210	178
Candidates enrolled	131	121	126	159	78	101	84	68
Enrolled/total ratio	9%	7%	9%	7%	44%	58%	40%	38%
Minimum entry score	68%	69%	67%	64%	17%	22%	22%	37%
Baseline weight factors	1	1.3	1	1.3	1.33	1	1.46	1.53

*Note*: The baseline weight factors were obtained by calculating the ratio of enrolled candidates to total candidates. Cohorts with the highest ratio received a weight factor value of 1, and cohorts with a lower ratio received weight factors that were adjusted accordingly. PD and ED groups were calculated separately.

To capture students' perceptions of the rapid shift to online learning, a survey was conducted twice, at the end of the 2019–2020 (Round 1) and 2020–2021 (Round 2) academic years, using Google Forms (Google LLC, CA, USA). Separate Polish and English versions were distributed to PD and ED students. Notably, survey participants in Round 1 began the academic year in person and then transitioned to online delivery, while those in Round 2 started online and then resumed in‐person classes. Because survey timing did not align perfectly with examination score groups (i.e., 2019C, 2020–21, and 2022) and the survey was anonymous, it was not possible to link participants' survey responses to specific official examination scores. Instead, the survey included self‐reported final anatomy examination status (“1 = exempted from the final examination,” “2 = passed in the first term,” “3 = retake,” “4 = did not pass yet”) as a proxy outcome of examination performance. This approach ensured participant privacy while still allowing the exploration of how learning resources and pandemic‐related impediments might correlate with examination performance.

The survey questionnaire comprised two parts. The first included a set of closed‐answer categorical questions to investigate the prevalent types of communication preferences, learning resources, and preferences for practical classes and examinations. It also explored time spent on online learning compared with in‐person semesters using a four‐point Likert‐type scale with no neutral option^50^ (“much less,” “less,” “more,” and “much more”).

Items in the communication category aimed at understanding the extent of the range of contacts maintained by students and their preferred contact methods. Additionally, technical issues related to internet connections or operating the applications were explored.

The classes and examination preferences category focused on students' preferred locations for theoretical and practical course activities, comparing home/dormitory and department environments. This category also aimed to determine the influence of having no access to the prosected specimens on online learning.

Participants were asked if virtual, photorealistic 3D specimens would affect their anatomy learning. The feedback collected with this question was intended to reinforce the discussion on the prospective introduction of virtual learning.^51^

The final category of the first part of the survey aimed to establish which learning resources students found most helpful in preparing for the examinations.

The second part of the survey included a set of four‐point Likert‐type scale (no neutral option) questions designed to measure the extent of problems experienced during online learning. Responses were “1 = no problem,” “2 = some problems,” “3 = moderate problems,” and “4 = serious problems.”

The survey items are presented in Table 5. The items were designed by anatomy professors, verified by the head of the department, and tested for construct validity using principal component analysis.^52^ Survey reliability was expressed as inter‐item consistency, measured using Cronbach's alpha.

**TABLE 5:** Survey questionnaire items and response options.

	Survey item	Answer options
1	Did you pass your anatomy exam in	Term “0,” First term, Retake, I have not passed yet
2	During the COVID‐19 pandemic, were you in touch with students from the entire first year?	Yes, No
3	During the COVID‐19 pandemic, were you only in contact with the students of your classes group?	Yes, No
4	During the COVID‐19 pandemic, were you in contact with an assistant conducting anatomy classes?	Yes, No
5	How did you stay in touch with other students during the COVID‐19 pandemic?	Phone, Messenger, Microsoft Teams, face‐to‐face
6	Regarding the remote learning during the COVID‐19 pandemic, did you have any problems with:	Internet connection, Moodle website, Microsoft Teams application
7	How much time did you spend learning anatomy in the online semester compared to the in person one?	Much less, Less, More, Much More
8	Where do you think writing the theoretical test is most convenient?	At home/In the dormitory, In the department, It does not matter
9	Where do you think writing the practical (spotters) test is most convenient?	At home/In the dormitory, In the department, It does not matter
10	In what way did the lack of direct contact with the prosected specimens affect your learning of Anatomy?	Made learning much easier, Made learning somewhat easier, Did not affect the learning, Made learning somewhat harder, Made learning significantly harder
11	What resources did you use for learning while preparing for the theoretical tests?	Textbooks, Online lectures, YouTube videos
12	What resources did you use for learning while preparing for the practical (spotters) tests?	Textbooks, Online lectures, ANKI, Specimen images, Department videos, YouTube videos
13	Do you think that virtual professionally made and easily accessible, realistic and interactive 3D preparations featuring free viewing from every angle and displaying the structures:	Won't improve the study of anatomy, Would improve my anatomy learning a little, Would greatly improve my anatomy learning
14	In connection with the COVID‐19, to what extent does each of the following statements pose a problem to you? Changing the form of academic classes to e‐learning Inability to travel Maintaining sufficient motivation to learn Accommodation situation (e.g., threat of closing the dormitory) No possibility of meeting friends, the need for physical isolation Postponement of credits/examinations Risk of extending the academic semester The general uncertainty of tomorrow The need to change my daily routine Threat to health of my loved ones Threat to my own health	No problems, Some problems, Moderate problems, Serious problems

### Data analysis

Examination scores and survey data were analyzed using IBM SPSS Statistics 28 (IBM Corp., NY, USA). The significance of all conducted statistical procedures was defined at the level of *α* = 0.05. Wherever multiple comparisons were used, p‐values were presented as adjusted, following the Bonferroni correction.

Examination scores were weighted for each cohort using normalized values from the enrolled‐to‐total candidates ratio (Table 4). The obtained weight factors were used for all subsequent score analyses.

The scores of the PD and ED groups were compared for each examination and each group separately, using the Mann–Whitney *U* test to determine whether to treat PD and ED students as single or separate groups. The Mann–Whitney *U* test compared male and female scores for each examination and group to identify any differences and adjust for them if found.

Next, examination scores were compared across the 2019C, 2020–21, and 2022 groups. Initially, the mean scores of all theoretical and practical examinations were compared using the Kruskal–Wallis test, followed by Dunn's test^53^ with Bonferroni correction for multiple comparisons to indicate which specific groups differed significantly from others. Subsequently, Kruskal–Wallis and Dunn's tests were performed for each examination separately across cohorts. The Kruskal–Wallis test was chosen over parametric variance analysis tests because the examination scores did not follow a normal distribution, as determined by the Kolmogorov–Smirnov test.

The effect size was provided with Epsilon square (*ε* ^2^), given that 0.04 ≤ *ε* ^2^ ≤ 0.15 corresponded to a moderate, 0.16 ≤ *ε* ^2^ ≤ 0.36 to a relatively strong, and *ε* ^2^ > 0.36 to a strong effect size.^54^

Passing rates were obtained by transforming the examination score dataset: Scores below 70% were marked as fail (0) and 70% or above as pass.^1^ The pass‐to‐fail frequencies were then compared with Bonferroni‐adjusted pairwise z‐tests for column proportions across the groups, separately for each examination.

Descriptive statistics were applied to analyze the frequency of survey answers. Chi‐square tests were conducted to study the differences in answer frequencies between 2019 and 2020 (Round 1) and 2020 and 2021 (Round 2). Likert‐scale question answers were expressed as means, and the interquartile range (IQR) was provided. Questions regarding using learning resources were dichotomized (0 = not used, 1 = used).

The validity of the questionnaire was assessed using principal component analysis (PCA). The suitability of the PCA was determined by examining the Keiser–Meyer–Olkin (KMO) measure of sampling adequacy and Bartlett's test of sphericity. The number of factors to be retained was based on fulfilling Kaiser's criterion (factor eigenvalues >1) and visually assessing the scree plot as a supplementary check. Varimax rotation was applied to clarify the mapping of survey items to the extracted factors. The survey items were also subjected to reliability analysis expressed as Cronbach's alpha, with values >0.7 indicating good internal consistency.

Additionally, linear regression was employed to elucidate the factors affecting the final anatomy examination performance. Likert‐scale learning impediment items and learning resources were entered as independent variables (predictors), and final anatomy examination performance (derived from the survey question) was entered as the dependent variable. Notably, the dependent variable was reverse‐coded; thus, an increase in the predictor value reflected a reduced risk of failure in the final examination. The best subset method was selected in the regression, and the adjusted *R* ^2^ was used as the predictor entry/removal criterion.

## RESULTS

### Examination scores and passing rates

The examination performance of the PD and ED groups differed significantly in 34 of the 48 compared examinations (eight theoretical and eight practical for each of the 2019C, 2020–21, and 2022 groups), as demonstrated by the Mann–Whitney *U* test (Table 6) and resulting in a decision to conduct separate analyses of these groups. In line with a previous study,^55^ there were no significant differences in scores between the majority of male and female students (Table 7). Therefore, male and female students' scores were pooled in the next steps of the analysis.

**TABLE 6:** Differences in the examination scores between Polish Division and English Division groups, as measured by the Mann–Whitney *U* test.

Polish Division vs. English Division		2019C		2020–21		2022	
		*U*	*p*	*U*	*p*	*U*	*p*
Theoretical block	Axial skeleton	2081.5	0.103	1437	<0.001	478	<0.001
	CNS	1763.5	<0.001	1757	<0.001	361	<0.001
	Head	2018	0.002	2587.5	0.061	438	<0.001
	Neck	1013	<0.001	571	0.002	422.5	<0.001
	Thorax	626.5	<0.001	3057.5	0.109	1704	<0.001
	Abdomen	694	<0.001	2874.5	0.021	988.5	<0.001
	Pelvis	2529	0.064	3269.5	0.29	943	<0.001
	Lower limb	1687	<0.001	3006	0.076	670.5	<0.001
Practical block	Axial skeleton	1302.5	<0.001	1665	0.076	1734.5	0.232
	CNS	2327.5	0.115	1617	<0.001	1203.5	0.159
	Head	1664	<0.001	2037	0.047	1225	0.001
	Neck	2604	0.215	718	0.319	1961	0.45
	Thorax	1572.5	<0.001	1819.5	<0.001	2192.5	0.011
	Abdomen	1783.5	0.011	2030	<0.001	2926.5	0.935
	Pelvis	1836	0.005	532.5	<0.001	1964	0.011
	Lower limb	2358.5	0.03	2389.5	<0.001	960.5	<0.001

**TABLE 7:** Examination score differences by gender, as measured by the Mann–Whitney *U* test.

			2019C		2020–21		2022	
			*U*	*p*	*U*	*p*	*U*	*p*
Polish Division	Theoretical block	Axial skeleton	761	0.09	776	0.947	1086.5	0.409
		CNS	782.5	0.034*	857	0.614	418.5	0.52
		Head	1071.5	0.391	920.5	0.797	1205	0.898
		Neck	1192.5	0.872	302.5	0.407	1170	0.901
		Thorax	1415.5	0.166	741	0.799	1516.5	0.914
		Abdomen	1299.5	0.527	673.5	0.432	1436	0.737
		Pelvis	1064	0.256	619.5	0.162	1460	0.362
		Lower limb	1038	0.231	588	0.182	1302.5	0.455
	Practical block	Axial skeleton	672	0.039*	679	0.929	1004	0.251
		CNS	864	0.23	666.5	0.079	368	0.416
		Head	1018	0.308	625	0.03*	973.5	0.635
		Neck	1039	0.285	326.5	0.951	1147	0.863
		Thorax	1242.5	0.814	756.5	0.914	1352.5	0.341
		Abdomen	1078.5	0.43	532	0.032	1396.5	0.551
		Pelvis	865.5	0.188	578	0.089	1204.5	0.584
		Lower limb	1272.5	0.535	583.5	0.199	1279	0.766
English Division	Theoretical block	Axial skeleton	262	0.118	325.5	0.93	135	0.747
		CNS	410	0.718	406	0.343	193.5	0.55
		Head	249	0.043*	363	0.034*	131.5	0.094
		Neck	389	0.874	110.5	0.785	177.5	0.511
		Thorax	340.5	0.353	696	0.565	244.5	0.378
		Abdomen	298.5	0.849	680.5	0.275	242	0.638
		Pelvis	336.5	0.245	768.5	0.912	169.5	0.243
		Lower limb	313.5	0.134	719.5	0.474	182.5	0.395
	Practical block	Axial skeleton	349.5	0.993	259	0.323	117	0.444
		CNS	416.5	0.781	222	0.079	210	0.852
		Head	450	0.087	309	0.311	168	0.8
		Neck	472.5	0.241	79	0.42	156	0.359
		Thorax	426.5	0.662	748.5	0.659	267.5	0.807
		Abdomen	278.5	0.62	710	0.57	231.5	0.313
		Pelvis	311	0.6	739	0.798	198.5	0.537
		Lower limb	389	0.874	739.5	0.597	158	0.399

*Note:* *Significant at *p* < 0.05.

**FIGURE 2:**
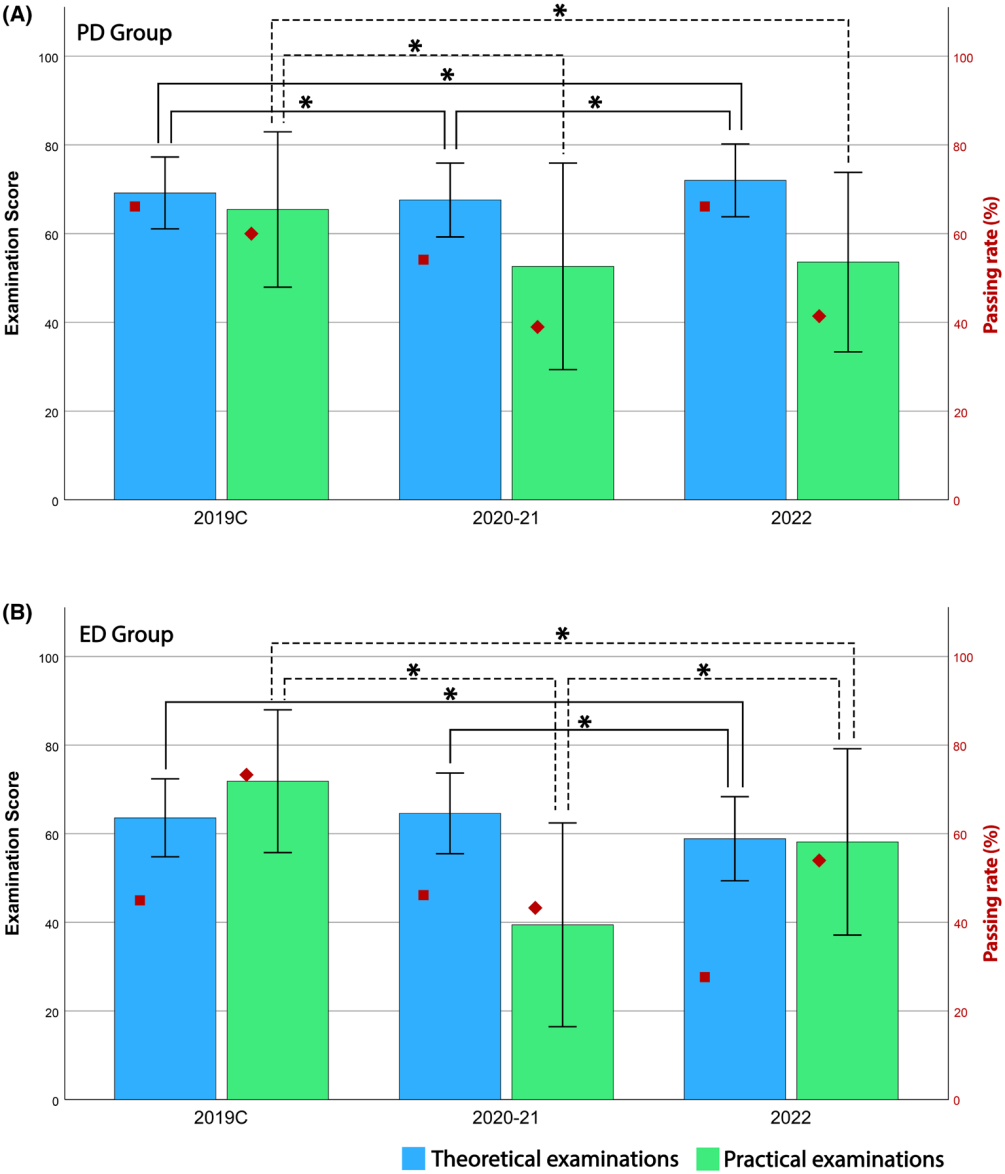
Examination scores comparison. Comparison of the examination scores between pre‐pandemic control group (2019C) and two comparison groups: Online (2020–21) and post‐lockdown (2022). (A) Comparison of PD students; (B) comparison of ED students. All Kruskal–Wallis results were significant at *p* < 0.001. Specifically, for PD students the theoretical part resulted in H[2] = 107,420 (*N* = 2339) and the practical part resulted in H[2] = 172,058 (*N* = 2280). For ED students, Kruskal–Wallis tests resulted in H[2] = 83,709; *N* = 1352, and H[2] = 408,923; *N* = 1312 for theoretical, and practical parts, respectively. All examination units were used in this comparison to achieve the overview of performance changes, which contributed to *N* values. *Difference was significant at *p* < 0.001.

In the PD cohorts, the mean theoretical score decreased slightly from 69.1 (SD ±7) in 2019C to 67.8 (SD ±7.9) in 2020–21, then rose to 72 (SD ±7.8) in 2022. By contrast, the mean practical score dropped from 65.2 (SD ±15.7) in 2019C to approximately 53 in 2020–21 and 2022, as shown in Figure 2A. In line with these shifts, the mean passing rate for theoretical exams changed by −11.94% in 2020–21 compared with 2019C and remained essentially unchanged (−0.18%) in 2022. For practical exams, mean passing rates remained lower than 2019C by −21.25% and by −18.6% in 2020–21 and 2022, respectively (Figure 2A).

In the ED cohorts, the mean theoretical score was 63.6 (SD ±8.2) in 2019C, then reached 64.4 (SD ±8.5) in 2020–21 before dropping to 58.8 (SD ±9) in 2022. The practical score changes were particularly pronounced as the mean practical score fell from 71.7 (SD ±14.1) in 2019C to 40.5 (SD ±20.2) in 2020–21 and partially rebounded to 57.8 (SD ±19.6) in 2022, as shown in Figure 2B. The mean passing rate for theoretical exams changed by −1.3% in 2020–21 relative to 2019C and by −27.6% in 2022. For practical exams, mean passing rates were −30.14% in 2020–21 and −18.43% in 2022 compared with 2019C (Figure 2B).

Regarding the specific examinations, theoretical examination score comparisons displayed a mixed pattern of increase and decrease depending on the examination unit. By contrast, the practical examinations showed a consistent reduction of scores across all significant comparisons for both the PD and ED groups. Per‐unit examination score comparisons are provided in Table 8A (PD students) and in Table 8B (ED students), and per‐unit breakdowns of students' passing rates are given in Tables 9A (PD) and 9B (ED).

**TABLE 8A:** Examination scores and pairwise comparisons across Polish Division student groups.

PD		Descriptive statistics									Pairwise comparisons			Kruskal–Wallis		Effect size (*ε* ^2^)
		2019C			2020–21			2022			2019C vs. 2020–21	2019C vs. 2022	2020–21 vs. 2022			
		*N*	Mean	SD	*N*	Mean	SD	*N*	Mean	SD	Diff.	Diff.	Diff.	H(2)	*N*	
Theoretical block	Axial skeleton	90	65.46	7.7	85	70.39	8.23	105	70.0	8.1	2020–21 > 2019C**	2022 > 2019C*	2020–21 > 2022	16.68	280	0.06
	CNS	94	60.28	8.03	95	63.04	6.68	68	72.1	6.8	2020–21 > 2019C	2022 > 2019C**	2022 > 2020–21**	73.05	257	0.29
	Head	100	68.64	7.19	96	65.77	7.62	106	74.7	9.4	2019C > 2020–21*	2022 > 2019C**	2022 > 2020–21**	58.62	302	0.19
	Neck	99	73.69	7.69	60	70.58	8.34	105	69.9	8.4	2019C > 2020–21*	2019C > 2022**	2020–21 > 2022	13.35	264	0.05
	Thorax	101	71.36	6.69	88	69.83	8.28	120	68.8	7.1	2019C > 2020–21	2019C > 2022*	2020–21 > 2022	6.89	309	0.02
	Abdomen	101	71.33	6.31	87	66.52	7.61	119	71.3	8.1	2019C > 2020–21**	2019C > 2022	2022 > 2020–21**	22.28	307	0.07
	Pelvis	102	68.52	5.32	88	70.14	9.13	125	73.1	6.5	2020–21 > 2019C*	2022 > 2019C**	2022 > 2020–21	30.94	315	0.1
	Lower limb	101	73.23	6.91	86	65.99	7.48	118	76.1	8.0	2019C > 2020–21**	2022 > 2019C*	2022 > 2020–21**	74.98	305	0.25
Practical block	Axial skeleton	87	49.17	18.35	85	45.41	20.19	105	51.21	21.13	2019C > 2020–21	2022 > 2019C	2022 > 2020–21	3.61	272	0.01
	CNS	93	66.05	18.79	95	39.52	18.9	68	52.23	21.13	2019C > 2020–21**	2019C > 2022**	2022 > 2020–21*	67.57	251	0.26
	Head	99	61.14	17.74	96	38.97	17.98	106	53.13	19.66	2019C > 2020–21**	2019C > 2022*	2022 > 2020–21**	57.78	301	0.19
	Neck	100	73.88	11.68	60	53.81	20.6	105	50.36	17.89	2019C > 2020–21**	2019C > 2022**	2020–21 > 2022	92.88	263	0.35
	Thorax	101	70.27	13.28	88	58.18	20.81	120	55.04	16.76	2019C > 2020–21**	2019C > 2022**	2020–21 > 2022	43.42	308	0.14
	Abdomen	100	74.35	15.46	87	72.2	18.8	119	69.47	16.81	2019C > 2020–21	2019C > 2022	2020–21 > 2022	5.18	305	0.02
	Pelvis	94	60.94	16.67	87	70.35	17.07	125	55.11	17.40	2020–21 > 2019C**	2019C > 2022*	2020–21 > 2022**	48.45	306	0.16
	Lower limb	100	65.42	13.94	85	43.88	22.75	118	40.16	19.59	2019C > 2020–21**	2019C > 2022**	2020–21 > 2022	73.19	303	0.24

*Note*: “Diff.” means direction of change in examination scores between the compared groups. The Kruskal–Wallis *N* values reflect weighted cases; therefore, the total *N* may differ from the sum of individual cohort *N*s. Significance is marked with * for *p* < 0.05, and with ** for *p* ≤ 0.001. Effect size values of 0.04 ≤ *ε* ^2^ ≤ 0.15 correspond to moderate, 0.16 ≤ *ε* ^2^ ≤ 0.36 to relatively strong, and *ε* ^2^ > 0.36 to strong effect size.^54^

**TABLE 8B:** Examination scores and pairwise comparisons across English Division student groups.

ED		Descriptive statistics									Pairwise comparisons			Kruskal–Wallis		Effect size (*ε* ^2^)
		2019C			2020–21			2022			2019C vs. 2020–21	2019C vs. 2022	2020–21 vs. 2022			
		*N*	Mean	SD	*N*	Mean	SD	*N*	Mean	SD	Diff.	Diff.	Diff.	H(2)	*N*	
Theoretical block	Axial skeleton	55	67.42	7.84	53	64.30	9.34	39	57.23	6.89	2019C > 2020–21	2019C > 2022**	2020–21 > 2022**	49.77	238	0.34
	CNS	59	65.12	8.50	62	57.90	8.34	44	58.20	8.68	2019C > 2020–21**	2019C > 2022**	2022 > 2020–21	29.52	270	0.18
	Head	56	62.35	10.39	65	63.75	8.17	40	57.90	9.18	2020–21 > 2019C	2019C > 2022**	2020–21 > 2022*	19.73	267	0.12
	Neck	59	61.02	10.30	31	65.35	5.89	42	53.29	8.24	2020–21 > 2019C	2019C > 2022**	2020–21 > 2022**	55.93	205	0.43
	Thorax	59	58.90	6.60	83	67.25	10.28	30	61.84	10.10	2020–21 > 2019C**	2022 > 2019C	2020–21 > 2022**	25.13	240	0.15
	Abdomen	48	60.75	7.08	82	63.83	8.73	49	58.51	9.68	2020–21 > 2019C	2019C > 2022	2020–21 > 2022**	19.04	232	0.11
	Pelvis	54	65.75	6.82	81	69.05	7.54	45	60.89	9.92	2020–21 > 2019C*	2019C > 2022*	2020–21 > 2022**	36.44	232	0.2
	Lower limb	59	67.40	7.88	83	63.96	8.52	45	62.16	9.55	2019C > 2020–21	2019C > 2022*	2020–21 > 2022	12.05	233	0.06
Practical block	Axial skeleton	55	65.73	21.94	53	52.06	20.33	39	46.78	18.78	2019C > 2020–21**	2019C > 2022**	2020–21 > 2022	27.79	233	0.19
	CNS	59	70.72	17.25	62	29.33	17.79	44	45.40	23.57	2019C > 2020–21**	2019C > 2022**	2022 > 2020–21**	93.26	251	0.57
	Head	56	72.59	16.73	65	44.55	17.46	40	64.61	17.41	2019C > 2020–21**	2019C > 2022*	2022 > 2020–21**	79.73	242	0.5
	Neck	59	76.36	11.20	31	48.39	20.77	42	53.90	21.62	2019C > 2020–21**	2019C > 2022**	2022 > 2020–21	61.38	197	0.47
	Thorax	59	81.44	10.82	83	38.61	20.16	50	61.89	19.53	2019C > 2020–21**	2019C > 2022**	2022 > 2020–21**	115.95	240	0.61
	Abdomen	48	68.59	14.90	82	54.48	23.20	49	67.65	19.23	2019C > 2020–21*	2019C > 2022	2022 > 2020–21**	17.43	230	0.1
	Pelvis	54	69.07	13.01	81	25.48	20.46	45	60.22	18.81	2019C > 2020–21**	2019C > 2022*	2022 > 2020–21**	101.08	227	0.56
	Lower limb	59	69.07	15.26	83	31.23	21.74	45	62.20	17.54	2019C > 2020–21**	2019C > 2022**	2022 > 2020–21	93	226	0.5

*Note*: “Diff.” means direction of change in examination scores between the compared groups. The Kruskal–Wallis *N* values reflect weighted cases; therefore, the total *N* may differ from the sum of individual cohort *N*s. Significance is marked with * for *p* < 0.05, and with ** for *p* ≤ 0.001. Effect size values of 0.04 ≤ *ε* ^2^ ≤ 0.15 correspond to moderate, 0.16 ≤ *ε* ^2^ ≤ 0.36 to relatively strong, and *ε* ^2^ > 0.36 to strong effect size.^54^

**TABLE 9A:** Examination passing rates across the Polish Division groups.

PD		Descriptive statistics						Pairwise comparisons		
		2019C		2020–21		2022		2019C vs. 2020–21	2019C vs. 2022	2020–21 vs. 2022
		First term (%)	Final (%)	First term (%)	Final (%)	First term (%)	Final (%)	Diff.	Diff.	Diff.
Theoretical block	Axial skeleton	54.0	92.2	58.8	93.3	52.4	91.0	2020–21 > 2019C	2019C > 2022	2020–21 > 2022
	CNS	26.6	91.2	29.5	81.0	64.7	91.0	2020–21 > 2019C	2022 > 2019C**	2022 > 2020–21**
	Head	78.0	98.0	52.1	82.9	73.6	88.0	2019C > 2020–21**	2019C > 2022	2022 > 2020–21*
	Neck	80.8	95.1	60.0	77.1	54.3	91.7	2019C > 2020–21*	2019C > 2022**	2020–21 > 2022
	Thorax	84.2	93.1	67.0	89.5	57.5	94.7	2019C > 2020–21*	2019C > 2022**	2020–21 > 2022
	Abdomen	72.3	96.1	51.7	90.5	63.9	91.0	2019C > 2020–21*	2019C > 2022	2022 > 2020–21
	Pelvis	47.1	88.2	71.6	95.8	80.0	95.5	2020–21 > 2019C**	2022 > 2019C**	2022 > 2020–21
	Lower limb	85.1	99.0	41.9	95.8	83.1	93.2	2019C > 2020–21**	2019C > 2022	2022 > 2020–21**
Practical block	Axial skeleton	17.2	93.1	18.8	86.7	39.0	94.7	2020–21 > 2019C	2022 > 2019C*	2022 > 2020–21*
	CNS	55.9	95.1	11.8	81.9	38.5	83.5	2019C > 2020–21**	2019C > 2022	2022 > 2020–21*
	Head	50.5	95.1	12.0	84.8	42.4	89.5	2019C > 2020–21**	2019C > 2022	2022 > 2020–21**
	Neck	81.0	98.0	40.7	87.6	28.8	84.2	2019C > 2020–21**	2019C > 2022**	2020–21 > 2022
	Thorax	72.3	99.0	52.3	92.6	37.8	91.0	2019C > 2020–21*	2019C > 2022**	2020–21 > 2022
	Abdomen	80.0	96.1	70.1	88.4	75.4	94.7	2019C > 2020–21	2019C > 2022	2022 > 2020–21
	Pelvis	53.2	88.2	79.3	98.9	50.0	94.7	2020–21 > 2019C**	2019C > 2022	2020–21 > 2022**
	Lower limb	72.0	98.0	27.1	90.5	21.4	99.2	2019C > 2020–21**	2019C > 2022**	2020–21 > 2022

*Note*: “Diff.” means direction of change in passing rates between compared groups. “First term” denotes passing rate in the first examination. “Final” denotes passing rate in the examination after a maximum of two retakes. Pairwise comparisons refer to the first term of examinations. Significance is marked with * for *p* < 0.05, and with ** for *p* ≤ 0.001.

**TABLE 9B:** Examination passing rates across the English Division groups.

ED		Descriptive statistics						Pairwise comparisons		
		2019C		2020–21		2022		2019C vs. 2020–21	2019C vs. 2022	2020–21 vs. 2022
		First term (%)	Final (%)	First term (%)	Final (%)	First term (%)	Final (%)	Diff.	Diff.	Diff.
Theoretical block	Axial skeleton	61.8	93.3	60.4	84.6	5.1	91.4	2019C > 2020–21	2019C > 2022**	2020–21 > 2022**
	CNS	60.3	95.0	17.7	91.0	27.3	93.1	2019C > 2020–21**	2019C > 2022**	2022 > 2020–21
	Head	38.6	93.3	50.8	93.6	30.0	96.6	2020–21 > 2019C	2019C > 2022	2020–21 > 2022*
	Neck	45.8	98.3	54.8	91.0	14.3	98.3	2020–21 > 2019C	2019C > 2022**	2020–21 > 2022**
	Thorax	16.9	95.0	59.3	90.4	38.0	89.7	2020–21 > 2019C**	2022 > 2019C*	2020–21 > 2022*
	Abdomen	23.5	98.3	44.6	88.3	30.6	93.1	2020–21 > 2019C*	2022 > 2019C	2020–21 > 2022
	Pelvis	60.0	91.7	57.3	88.3	31.1	93.1	2019C > 2020–21	2019C > 2022*	2020–21 > 2022*
	Lower limb	51.7	88.3	24.1	87.2	44.4	96.6	2019C > 2020–21*	2019C > 2022	2022 > 2020–21*
Practical block	Axial skeleton	70.9	96.7	31.4	89.7	26.3	93.1	2019C > 2020–21**	2019C > 2022**	2020–21 > 2022
	CNS	69.5	96.7	9.6	92.3	34.1	91.4	2019C > 2020–21**	2019C > 2022**	2022 > 2020–21**
	Head	71.4	91.7	18.2	91.0	76.3	93.1	2019C > 2020–21**	2022 > 2019C	2022 > 2020–21**
	Neck	86.4	98.3	39.3	94.9	46.3	89.7	2019C > 2020–21**	2019C > 2022**	2022 > 2020–21
	Thorax	89.8	98.3	59.0	88.3	63.3	89.7	2019C > 2020–21**	2019C > 2022*	2022 > 2020–21**
	Abdomen	62.5	95.0	43.9	92.6	70.0	91.4	2019C > 2020–21	2022 > 2019C	2022 > 2020–21**
	Pelvis	66.7	91.7	84.0	95.7	58.7	94.8	2020–21 > 2019C**	2019C > 2022	2020–21 > 2022**
	Lower limb	69.5	93.3	60.2	88.3	64.3	98.3	2019C > 2020–21**	2019C > 2022	2022 > 2020–21**

*Note*: “Diff.” means direction of change in passing rates between compared groups. “First term” denotes passing rate in the first examination. “Final” denotes passing rate in the examination after a maximum of two retakes. Pairwise comparisons refer to the first term of examinations. Significance is marked with * for *p* < 0.05, and with ** for *p* ≤ 0.001.

### Exploring the survey responses

Across the four survey sets (two rounds × two student groups), 11 of the 36 items demonstrated significant gender differences (Table 10). However, no more than four such differences were observed within any single set, and the pattern between rounds and divisions was inconsistent. Effect sizes were generally small to moderate. Given the inconsistency of these findings and the uneven gender distribution, the remaining survey analyses were conducted with genders combined.

**TABLE 10:** Survey items showing significant gender differences across student divisions and survey rounds.

Survey item	PD Round 1			PD Round 2			ED Round 1			ED Round 2		
	*U*	*p*	|*r*|	*U*	*p*	|*r*|	*U*	*p*	|*r*|	*U*	*p*	|*r*|
Staying in touch with classmates							92.5	0.016	0.46	158	0.031	0.31
Phone call contact										308.5	0.013	0.35
Face‐to‐face contact	379.5	0.013	0.3							312.5	0.014	0.37
Convenient place for the theoretical examination							93.5	0.017	0.45			
Convenient place for the practical examination										285	0.021	0.25
No access to prosected specimens				529.5	0.048	0.21						
Learning resources for online practical examinations: Department videos				561.5	0.016	0.17						
Postponement of credits/exams							243	0.029	0.42			
The need to change one's daily routine							245.5	0.022	0.44			
Threat to health of loved ones	161.5	0.003	0.45	475	0.013	0.3						
Threat to one's health	151.5	0.005	0.48	362.5	0.001	0.46						

*Note*: |*r*| = absolute value of effect size estimated by rank‐biserial correlation (based on Mann–Whitney *U* test). Only items with *p* < 0.05 are shown. Given the inconsistent pattern of the differences across survey rounds and student groups, these results should be interpreted cautiously, as isolated significant findings may reflect chance variation rather than consistent trend.

The PCA retained nine factors explaining 63.21% of the variance, supported by a KMO value of 0.612 and a significant Bartlett's test (*χ* ^2^ = 1223.06, *p* < 0.001). Each factor measured a distinct construct, indicating adequate survey validity, though Cronbach's alpha (*α* = 0.685) was slightly below the recommended level^56^ of *α* ≤ 0.7, possibly due to item heterogeneity. The distribution of survey items across the retained factors is shown in Table 11.

**TABLE 11:** Results of principal component analysis performed on the survey items to assess their validity.

Survey item	Factor eigenvalue	Percent of variance explained	Factor loading
Accommodation situation (e.g., threat of closing the dormitory)	3.24	12.47	0.46
The general uncertainty of tomorrow			0.73
Threat to health of loved ones			0.74
Threat to one's health			0.86
Changing the form of academic classes to e‐learning	2.77	10.65	0.49
Inability to travel			0.47
Maintaining sufficient motivation to learn			0.57
No possibility of meeting friends, the need for physical isolation			0.6
The need to change one's daily routine			0.69
Theoretical learning: online lectures	2.11	8.12	0.76
Practical learning: online lectures			0.77
Theoretical learning: YouTube videos			0.55
Practical learning: specimen images			0.49
Postponement of credits/examinations	1.86	7.14	0.81
Risk of extending the academic semester			0.76
Theoretical learning: textbooks	1.54	5.93	0.72
Practical learning: textbooks			0.77
Practical learning: department videos			0.74
Staying in touch with students from the entire first year	1.52	5.84	−0.83
Staying in touch with classmates			0.89
Preferred form of practical examination	1.26	4.84	−0.83
No access to prosected specimens			0.81
Convenient place for the practical examination			0.83
Convenient place for the theoretical examination			0.42
Moodle problems	1.11	4.26	0.78
Microsoft Teams problems			0.71
Internet connection problems	1.06	4.07	0.66

The survey was conducted in two rounds across 2020 and 2021, with 54 PD (female = 72%) and 37 ED (female = 49%) students in the first round, rising to 90 PD (female = 79%) and 43 ED (female = 56%) students in the second round. The absolute response rates were 57% for PD students and 39% for ED students in the first round, rising to 86% and 55% in the second round, respectively.

Round 1 of the survey explored students' attitudes toward midyear shifts from in‐person to remote learning. On the contrary, as Round 2 participants started their anatomy course online and reverted to the in‐person learning form in midyear, their opinions varied.

During online learning, the percentage of PD students studying primarily from home decreased significantly in Round 2 compared with Round 1 (*p =* 0.002). In Round 1, mixed responses were received from ED students, of whom 54.1% studied at home, while the remaining 45.9% studied either in a dormitory or rented an apartment. In Round 2, the percentage of ED students studying primarily from home also decreased significantly (*p =* 0.017).

#### Students' communication

In Round 2, PD students showed a significant increase in contact with other class members (*p* = 0.028). An increase was also observed among the ED students, although the difference was not significant. PD students reported a significant decrease in contact with other year's members in Round 2 compared with Round 1. Most PD students in both rounds preferred Messenger (Meta Inc.); however, its usage dropped significantly (*p =* 0.005) among ED students in Round 2 in favor of phone calls. The frequency of personal meetings increased from Round 1 to Round 2 among all students; however, this finding was not significant.

#### Practical classes and examination preferences

Unsurprisingly, a clear preference for in‐person practical examinations using prosected specimens was observed in Round 1 and increased further in Round 2. This preference was not present in the theoretical examinations. Access to prosected specimens was crucial, significantly easing learning. On average, nearly 73.1% of students in both divisions reported increased difficulty when specimens were unavailable.

In both survey rounds, most students expressed interest in the future introduction of virtual photorealistic specimens to support anatomy learning.

#### Time spent on learning

Transitioning to online learning led to an increased study time for anatomy among 94.4% of PD students in Round 1 of the survey. Unexpectedly, this finding was not found in Round 2 of the survey, with only 46.7% of PD students spending more time learning anatomy compared with the time spent on in‐person learning. This was not reflected in the ED groups: In Round 1, 89.2% of ED students spent more time learning online, and a similar result was observed in Round 2. Spending considerably more learning time during the pandemic was echoed in another study.^57^

#### Online learning impediments

The primary concern among PD students during both survey rounds was the “threat to the health of their families.” In Round 1, their second‐highest concern was the risk of semester extension, while in Round 2, social isolation became more important. The prevalent concern among the ED students during Round 1 was the risk of semester extension, shifting during Round 2 to moving online and the “general uncertainty of the future.” The accommodation situation was the issue of least concern to all survey participants.

Detailed survey item responses are presented in Table 12.

**TABLE 12:** Survey questionnaire items and participants' responses.

Survey item		Polish Division		English Division	
		Round 1 (2020)	Round 2 (2021)	Round 1 (2020)	Round 2 (2021)
*Communication*					
Maintaining social contact	Staying in touch with first‐year students	74.1%	45.6%	43.2%	53.5%
	Staying in touch with classmates	38.9%	57.8%	48.6%	69.8%
	Staying in touch with an assistant conducting/preparing anatomy classes	81.5%	77.8%	56.8%	67.4%
Methods of contact	Phone call contact	44.4%	30.0%	45.9%	69.8%
	Messenger contact	98.1%	100.0%	94.6%	69.8%
	Microsoft Teams contact	68.5%	27.8%	62.2%	37.2%
	Face‐to‐face contact	18.5%	31.1%	32.4%	37.2%
Technical issues	Internet connection problems	79.6%	87.8%	70.3%	95.3%
	Moodle‐related problems	55.6%	40.0%	91.9%	41.9%
	Microsoft Teams problems	44.4%	57.8%	37.8%	37.2%
*Time spent on learning online*					
Time spent on learning during the online semester, compared with stationary learning	Much less	0.0%	5.6%	0.0%	7.0%
	Less	5.6%	47.8%	10.8%	9.3%
	More	61.1%	31.1%	35.1%	46.5%
	Much more	33.3%	15.6%	54.1%	37.2%
*Classes and examination preferences*					
Convenient place for the theoretical examination	At home/In the dormitory	24.1%	47.8%	32.4%	11.6%
	It does not matter	48.1%	32.2%	54.1%	55.8%
	In the department	27.8%	20.0%	13.5%	32.6%
Convenient place for the practical examination	At home/In the dormitory	7.4%	1.1%	10.8%	7.0%
	It does not matter	5.6%	0.0%	16.2%	7.0%
	In the department	87.0%	98.9%	73.0%	86.0%
Preferred form of practical examination	Traditional (in the department dissection room)	87.0%	91.1%	89.2%	95.3%
	Online	13.0%	8.9%	10.8%	4.7%
No access to prosected specimens	Made learning significantly harder	57.4%	77.8%	75.7%	81.4%
	Made learning somewhat harder	42.6%	13.3%	13.5%	16.3%
	Did not affect the learning	0.0%	0.0%	8.1%	0.0%
	Made learning somewhat easier	0.0%	4.4%	0.0%	0.0%
	Made learning much easier	0.0%	4.4%	2.7%	2.3%
Potential usability of virtual 3D anatomy specimens	Will make it difficult to learn anatomy	0.0%	0.0%	2.7%	0.0%
	Will not improve the study of anatomy	5.6%	8.9%	5.4%	11.6%
	Will improve anatomy learning a little	44.4%	44.4%	59.5%	39.5%
	Will greatly improve anatomy learning	50.0%	46.7%	32.4%	48.8%
*Learning resources*					
Learning resources for online theoretical examinations	Textbooks	100.0%	98.9%	100.0%	98.9%
	Online lectures	79.6%	75.6%	79.6%	75.6%
	YouTube videos	59.3%	63.3%	59.3%	63.3%
Learning resources for online practical examinations	Textbooks	96.3%	92.2%	96.3%	92.2%
	ANKI	96.3%	98.9%	96.3%	98.9%
	Department videos	100.0%	92.2%	100.0%	92.2%
	YouTube videos	42.6%	50.0%	42.6%	50.0%
	Specimen images	70.4%	46.7%	70.4%	46.7%
	Online lectures	40.7%	30.0%	40.7%	30.0%
*Online learning impediments*					
Type of issues	Changing the form of academic classes to e‐learning	2.76	2.69	2.86	3.12
	Inability to travel	2.44	2.9	2.49	2.79
	Maintaining sufficient motivation to learn	2.94	2.78	2.78	2.86
	Accommodation situation (e.g., threat of closing the dormitory)	1.91	1.88	2.27	2.28
	No possibility of meeting friends, the need for physical isolation	2.81	3.21	2.7	2.88
	Postponement of credits/examinations	3.04	2.42	3.11	2.53
	Risk of extending the academic semester	3.11	2.86	3.14	2.74
	The general uncertainty of tomorrow	2.96	3	3.11	3.09
	The need to change one's daily routine	2.54	2.52	2.57	2.21
	Threat to health of loved ones	3.56	3.56	2.89	3.05
	Threat to one's health	2.83	3.03	2.62	2.79

*Note*: Values in the online learning impediments section represent mean participants' agreement level, according to a four‐point Likert scale. In this section of the survey, the interquartile range (IQR) was 2 except for “threat to health of their families” in the PD students' group (IQR = 1). Values in the remaining sections represent frequencies of the given answer.

### Impact of learning resources and pandemic impediments on final examination performance

Results of the following section rely on the self‐reported, survey‐based final anatomy examination outcomes rather than official departmental scores due to anonymity constraints in our survey design.

The linear regression examining how learning resources predicted final examination performance resulted in a poorly fitted model in the PD group (adj. *R* ^2^ = 0.07), with the use of instructional department videos found to reduce the risk of failure significantly. This finding was reproducible using Spearman's rank correlation, yielding a weak but significant negative association (*r* = −0.23, *p =* 0.006). Additionally, PD students who used instructional videos often also tended to use textbooks (*r* = 0.33, *p <* 0.001), which was also observed in the ED group (*r* = 0.49, *p <* 0.01). In the ED group, a small but considerable regression model was achieved (adj. *R* ^2^ = 0.3), clarifying that textbooks (*R* ^2^ = 0.22, *p =* 0.02) and anatomy videos found on YouTube (*R* ^2^ = 0.23, *p =* 0.02) reduced the risk of failing the final examination. These findings were partially supported by Spearman's rank correlations, with significant associations between reduced failure risk and learning from textbooks (*r* = −0.31, *p =* 0.006), YouTube videos (*r* = −0.43, *p <* 0.001), and online lectures (*r* = −0.3, *p =* 0.007).

When analyzing learning impediments, the regression model resulted in weak but possibly relevant influences on final examination performance (PD student model fit: adj. *R* ^2^ = 0.16; ED student model fit: adj. *R* ^2^ = 0.31). In the PD group, significant factors increasing the risk of failing were: “Postponements of credits/exams” (*R* ^2^ = 0.3, *p =* 0.03), “Maintaining sufficient motivation to learn” (*R* ^2^ = 0.25, *p =* 0.008), and “Need for social isolation” (*R* ^2^ = 0.23, *p =* 0.01). In the ED group, significant factors were “Need for social isolation” (*R* ^2^ = 0.4, *p* = 0.01), “Threat to the health of their families” (*R* ^2^ = 0.31, *p* = 0.004; higher concern = lower failure risk), and accommodation situation (*R* ^2^ = 0.17, *p* = 0.01).

## DISCUSSION

### Impact of the transition to online learning on students' examination performance

Practical examination scores and pass rates dropped sharply during the shift to online learning and remained depressed after the reassumption of in‐person classes, while theoretical scores changed little.

Restricted access to prosected specimens, motivational issues, technical difficulties, and the stress of online proctoring may all have contributed to the decline in scores.^28^, ^58^ Yet the persistence of lower practical scores across cohorts probably points to a deeper cause: weak constructive alignment in the curriculum, especially since no straightforward methods for translating constructive alignment into an online environment existed at the outbreak of COVID‐19. In constructive alignment, teaching, learning, and assessment are designed to match the intended learning outcomes. Those are typically stated upfront, and the students are encouraged to gain their knowledge as a result of their own learning activities.^59^

Because the 2022 group had completed most of their final high‐school years online, the study habits and engagement patterns shaped in that environment likely translated into their performance at the university,^60^ further amplifying the curriculum misalignment.

The pandemic‐related studies converge on a consistent set of curriculum design suggestions. First, programs that preserved constructive alignment—explicit mapping of digital tasks to the same spatial, clinical, and reasoning outcomes assessed in face‐to‐face spotters—showed the smallest performance losses.^18^, ^24^, ^30^ Second, a shift toward active, student‐centered learning—pre‐lab videos, structured peer teaching, and small‐group team‐based learning—was repeatedly linked to higher engagement and faster recovery of practical skills once on‐site sessions resumed.^57^, ^61^ Third, studies that embedded formative components—online quizzes and one‐on‐one feedback sessions—as a layer built on top of the in‐person practical classes mitigated anxiety and promoted intrinsic motivation.^38^, ^62^ Finally, the most resilient curricula maintained a hybrid resource ecosystem, pairing hands‐on prosection activities with digital assets (annotated photos, instructional videos) rather than attempting a total replacement of the in‐person, donor‐based classes.^6^, ^51^

Contrasting current findings with these principles suggests that the performance of online and post‐pandemic cohorts was undermined by an insufficient alignment and thin structure of formative activities; simply reverting to the pre‐COVID course program is not enough. The remedial steps—enhanced feedback sessions, 3D specimen atlas, reinforced flipped‐classroom activities—are aimed at strengthening practical competence in post‐pandemic cohorts.

Using the examination performance comparisons, H1–H3 have been evaluated as follows.

(Practical—lockdowns vs. pre‐pandemic): Accepted. In both PD and ED, practical mean scores and first‐term passing rates during 2020–21 were markedly below 2019C.

(Practical—post‐lockdowns): Accepted. Practical performance in 2022 remained below 2019C in both divisions (partial rebound in ED), with lower first‐term passing rates than pre‐pandemic.

(Theoretical): Across the compared student groups, changes in theoretical performance were smaller than those observed for practical examinations.

### The perspective on digital tools

The reliability of digital methods remains a subject of ongoing debate, even after pandemic conditions have eased, and a set of strengths and weaknesses of these methods has been documented. Particularly, instructional videos were evaluated as overly positive,^29^, ^63^, ^64^ and short (less than 5 min) videos were reported to be the most engaging.^65^ By contrast, some videos were linked to increased cognitive load.^66^ Digital images are a convenient method when the donor‐based examinations are costly to run, but some concerns regarding a clear orientation of the specimen represented on the image were raised, and spatial context was lost.^67^ 3D digital models, while innovative and preserving the spatial relationships, have been reported to contribute to lower results than in the hands‐on student group.^68^, ^69^ Finally, creating and implementing any digital content comes at the expense of faculty time.^70^

The integration of pre‐recorded, asynchronous instructional videos is advised as a valuable element of modern hybrid curricula, providing a balanced middle ground between static imagery and advanced 3D techniques. These videos offer considerable flexibility, enabling on‐demand access, pausing, rewinding, and targeted re‐viewing of key segments. Although reduced student engagement with video materials has been reported,^38^ this limitation can be mitigated by embedding interactive features, such as context‐specific questions that allow the video to resume only after the correct answer is selected, thereby sustaining attention and reinforcing learning outcomes.

### Student‐reported experiences

To complement the analysis of examination scores, an anonymous survey was conducted to gain insight into factors that may explain the deterioration in examination performance. Survey response rates were relatively low, especially in Round 1, compared with the minimum recently recommended 67%^71^ to reduce nonresponse bias and support convenient generalization.^71^, ^72^ Students who experienced fewer technological or motivational difficulties may have been more likely to respond. Conversely, those experiencing more pronounced challenges might be under‐represented. Thus, current data could underestimate the prevalence of impediments such as elevated anxiety levels, decreased motivation to learn, or technical constraints. The response rate improved in Round 2, possibly reflecting adaptation to pandemic‐related challenges after experiencing online learning for a whole semester and the resumption of in‐person classes. These limitations warrant cautious interpretation when comparing this study with others. Future surveys might deploy repeated reminders or covering to increase participation in medical education research.^73^

Regression analyses of self‐reported final anatomy examination outcomes (ranging from “exempted” to “did not pass yet”) revealed that instructional videos (in PD) and YouTube videos combined with textbooks (in ED) contributed to a reduced risk of failing the final examination. These findings suggest that digital resources—especially videos—can enhance students' preparedness. Reflecting the current results, summary pre‐lab videos have been considered an important component of the anatomy curriculum, which may contribute to improving learning performance.^63^, ^70^

By contrast, pandemic‐related learning impediments were associated with an increased risk of failure on the final anatomy examination. Concerns regarding education, examinations, and the successful finalization of the academic year were also observed in another study.^74^ Notably, the ED group reported more pronounced struggles, particularly a combination of accommodation concerns, health threats, the need for physical isolation, and general uncertainty about the future, as evidenced by the PCA analysis. These challenges could have contributed to elevated stress and anxiety levels in the ED students and remain in line with another study, where increased stress levels were found among international students, resulting from university campus closures, semester extensions, delays in examinations, and lack of appropriate learning environments.^75^ Elevated anxiety levels caused by social distancing have also been documented.^76^, ^77^

Another element leading to differences in the performances of the PD and ED groups would be much higher Moodle‐related technical problems reported by the latter, especially during Round 1 of the survey. This outcome was of concern, as Moodle is commonly used to deliver learning content and assessments. This concern could be mitigated by increasing the extent and quality of interactions between teachers and learners, as lack of interaction and technology issues were the most frequent problems reported during online education.^78^ Image‐based practical examinations and continuous proctoring may have elevated the anxiety levels of ED students, already coping with considerable pandemic‐related concerns, and eventually led to the deterioration of examination performance.

Although the examination scores and anonymous survey data come from separate analyses, both point to the critical role of effective curriculum design and student‐centered strategies. For instance, while the considerable use of departmental videos pointed to students' engagement with digital resources, the lowered examination performance revealed limitations not only when in‐person learning is absent but also when those resources are insufficiently aligned with examination expectations.

Using the regression analyses of the survey data, H4 and H5 have been evaluated as follows.

(Learning resources): Accepted. Greater use of structured resources—departmental instructional videos (PD) and textbooks/YouTube (ED)—was associated with a reduced self‐reported risk of failing the final examination.

(Learning impediments): Accepted. Higher levels of pandemic‐related impediments (e.g., low motivation, social isolation, postponements; in ED, also family‐health concerns and accommodation) were associated with an increased self‐reported risk of failure.

### Results relevance

As the misalignment between the instruction and the practical assessment was identified, current insights may guide educators who face the need to refine the curriculum and improve constructive alignment. Present data offer a clear baseline against which the effectiveness of future, better‐aligned courses can be benchmarked. Curriculum revising, now widely acknowledged as essential^10^, ^36^, ^79^, ^80^ in the long wake of COVID‐19, will likely benefit precisely from such reference points.

The outcomes of using the online resources described in this study—video tutorials and annotated images—show that merely adding digital tools to a curriculum is not enough; they must be carefully aligned with the core in‐person dissection‐ or prosection‐based activities so that both target the same learning outcomes.^81^ Taken together, these observations underscore that the pedagogical foundation still relies on well‐designed, face‐to‐face practical activities; therefore, any refinement of assessment practices must first optimize the in‐person format before digital enhancements can yield their full value.

The in‐person experiences—primarily face‐to‐face peer discussions and practical examinations—are currently irreplaceable,^38^ and pandemic‐related loss of these modalities may have a lasting impact on learning performance.^5^ This observation has been demonstrated in current survey results and thoroughly underscored in another study, where it was acknowledged that anatomical education is particularly tactile, sensory, and person‐centered.^82^ Such traits are unlikely to be achieved in an entirely digital learning environment; instead, the latter, as a vital component of a blended curriculum, may ease the steep curve of learning in a donor‐based, in‐person environment, eventually leading to increased confidence during practical examinations.

For example, implementing viva voce (oral) practical examinations can result in higher scores than the commonly used “bell‐ringer” style examinations.^83^ The latter disallows direct specimen handling, which does not reflect the laboratory‐class environment that heavily relies on manipulating specimens, potentially disadvantaging kinesthetic learners. Applying viva voce delivery would reflect the typical laboratory setup, encourage students to demonstrate a deeper anatomical understanding, and ensure inclusiveness for all types of learners, including those with kinesthetic, visual, and auditory learning styles. Students also report difficulty finding proper orientation in two‐dimensional images of anatomical structures during online examinations.^18^ This issue is far less pronounced when they can physically approach a specimen from multiple angles and assess spatial relationships in real time. Moreover, applying untimed practical examination questions may contribute to score improvement.^84^

Additionally, low‐performing students especially suffer from the lack of collaborative learning and feedback that occurs during peer discussions.^61^ One likely adverse effect of the pandemic was the hindered possibility of face‐to‐face discussions; hence, implementing structured peer discussions such as peer‐teaching activities, small‐group formative quizzes, or gamification elements like Anatomy Escape Room^85^ may increase examination confidence, underpinned by a deep understanding of anatomy.

Current results also demonstrate the psychological effects of the pandemic, which are felt most acutely by international students. These barriers—decreased engagement, anxiety linked to health concerns, and social isolation—may continue to undermine performance even after restrictions have ended.

Mechanisms for feedback should not be overlooked. Following the pandemic, one‐on‐one extended feedback sessions were implemented, in which students met with teaching staff to review their practical examination answers. Some students demonstrated a deeper understanding of anatomy than their scores suggested. Recurrent errors on specific specimens were traced to common misinterpretations rather than knowledge gaps. Such insights underscore that poor examination performance may stem from how students interpret questions. Notably, question types and formats possibly influence student performance.^86^ Moreover, LMS platforms (e.g., Moodle) often include robust item‐analysis statistics, particularly the facility index and discrimination index, which are recommended for evaluating the quality of examination questions.

Continuing the observation that digital methods alone are not enough, it is advised that curricular refinement should be prioritized over investments in more advanced technologies (for example, virtual reality or 3D digital platforms), whose development and maintenance are often both time‐ and cost‐intensive; in addition, the use of such technologies provides mixed results—for example, the preference toward the virtual specimens varied.^68^, ^87^ On the contrary, computer‐aided learning demonstrated good educational value when compared to its cost.^35^

Pre‐COVID pass rates in 2019C cohorts were already low, indicating pre‐existing curricular gaps, such as insufficient alignment of learning resources (lectures, textbooks, and atlases), peer discussion and review components, and structures assessed during practical examinations. To close this gap, formative, team‐based learning sessions should facilitate learning complex structures and promote collaborative problem‐solving.^62^, ^88^, ^89^ Moreover, team‐based learning enhanced students' performance in the summative assessments.^62^ Additionally, expanding flipped‐classroom hours will maximize hands‐on dissection time, while curated resources can prevent information overload commonly reported with textbooks and atlases.^90^

Post‐pandemic anatomy programs will thrive when digital resources, hands‐on activities, and student support mechanisms are integrated and continuously realigned with the intended learning outcomes.

### Future directions

The current study emphasizes the critical importance of a well‐constructed and up‐to‐date curriculum; however, its actual effectiveness needs to be confirmed across multiple institutions and contexts, with current results potentially contributing to a baseline comparison component. A successful attempt at course redesign has been documented.^91^ However, the authors of the cited study called for replication studies, and replicability plays a major role in educational research, as previously discussed.^92^ While the importance of a collaborative approach is already recognized,^93^ it is advised to continue shifting toward multi‐institutional evaluations of the anatomy programs to generalize best practices.

It is also suggested that future curricula should move beyond gross anatomy by integrating a clinical component, as this approach appears beneficial for enhancing long‐term knowledge retention and clinical competency, since the clinical aspect of anatomy necessitates frequent application in practice.^94^ By following student cohorts beyond the standard course activities, researchers can correlate anatomy learning outcomes with clinical performance, providing evidence on how well early anatomy training translates to practice. Conversely, failing to apply the clinical context impeded the transfer of anatomy learning.^95^

Embedding clinical relevance into gross anatomy training, increasing opportunities for formative assessments, and including flipped‐classroom strategies may help address the performance gap.

Additionally, future studies with larger, gender‐balanced cohorts may help determine whether the observed survey differences represent genuine trends. Such studies could provide insights into whether learning preferences and perceived challenges in anatomy education differ meaningfully between students of different genders.

### Limitations

This study covered a single university. Researcher bias might have occurred when trying to interconnect the anatomy course design, which is qualitative in nature, with quantitative student performance outcomes.

Sample sizes varied by examination, as the number of students allowed to take the first examination term depended on their performance in the class‐entrance tests.

Notably, the anatomy laboratory hours at the Department of Anatomy of Collegium Medicum UWM are roughly double the average 76‐h course duration in the United States,^96^ Australia, and New Zealand,^97^ making it difficult to directly compare the results of this study with other studies. Moreover, it is more common for students outside of the United States to enroll in medical school immediately after graduating high school, which further impedes the possibility of direct comparisons with other US‐based studies.

Another issue concerns students' initial performance. This was partially mitigated by weighting the examination scores according to baseline performance; however, the baseline was obtained with limited data.

Another limitation was the survey response rate, which was below the recently recommended level of 67%.^71^ Participants who felt strongly (positive or negative) may have been more likely to complete the survey,^98^ which may not have reflected the perceptions of the entire group and introduced the nonresponse bias as a result.^99^ Therefore, caution is recommended in generalizing current survey findings, mainly since the current study covered a single institution.

Additionally, the single‐item survey design could decrease reliability, and prospective studies in this area could benefit from improved multi‐item survey constructs.

Finally, the post‐pandemic course adaptations are preliminary, and their outcomes have not yet been researched. Therefore, it was not possible to evaluate the influence of these methods on student performance.

## CONCLUSION

This study compared anatomy examination performance during the COVID‐19 pandemic online learning with performance in the first year after returning to in‐person delivery (Primary Objective) and explored the self‐reported learning resources, examination preferences, and pandemic‐related learning impediments influencing those outcomes (Secondary Objectives).

Current results confirm that practical examination scores decreased significantly when classes shifted online and remained below pre‐pandemic levels even after in‐person course mode was resumed. On the contrary, theoretical examination scores were less affected. Further, survey findings revealed that students who willingly used departmental videos and other digital tools were less likely to fail the final anatomy examination. Additionally, motivational issues, social isolation, and possible misalignment between learning and examination contents emerged as risk factors.

In a broader context, the findings demonstrate that merely reusing a pre‐pandemic teaching and learning approach may no longer ensure the high performance standards. Instead, an adjustment of curriculum design—including a student‐centered approach, stronger formative components, and careful alignment of digital and hands‐on materials with the summative assessments—is needed to maintain a high educational standard. By blending^100^ the best practices from innovations introduced during the pandemic with a robust in‐person learning component, anatomy education can become more resilient, better aligned to intended learning outcomes, and ultimately more effective in preparing students for clinical practice.

## AUTHOR CONTRIBUTIONS

**Michał Ciucias:** Conceptualization; formal analysis; methodology; writing – original draft; writing – review and editing. **Katarzyna Polak‐Boroń:** Conceptualization; visualization; writing – original draft. **Marcelina Łopińska:** Conceptualization; visualization; writing – original draft. **Jerzy Stanisław Gielecki:** Conceptualization; project administration; supervision. **Anna Żurada:** Writing – review and editing.

## ETHICAL APPROVAL

This study was approved by the Scientific Research Ethics Committee of the University of Warmia and Mazury in Olsztyn, Poland (Decision No. 24/2021, 17 November 2021).

## Supporting information


**Table S1.** Example set of questions from a final unit theoretical examination.

**Table S2.** Example list of structures from a final unit practical examination.
