# Supplementary material for: Medical students' examinations during the pandemic: Performance and perceptions of anatomy teaching and learning before, during, and after COVID‐19 lockdowns
Source: Anat Sci Educ. 2025 Oct 16;19(1):46–71. doi: 10.1002/ase.70136 (PMC12748042; doi:10.1002/ase.70136)
Supplement: Supplementary file 1 — Table S1. Example set of questions from a final unit theoretical examination. [file ASE-19-46-s002.docx]

**Example set of questions from a final unit theoretical examination (central nervous system)**

| **Question** | **T/F** |
| --- | --- |
| 1. Which of the following venous sinuses are associated with confluence of sinuses: |  |
| Superior sagittal sinus | T |
| Occipital sinus | F |
| Inferior sagittal sinus | F |
| Straight sinus | T |
| Sigmoid sinus | F |
| 2. Indicate the proper statement(s). |  |
| Dura mater of the cerebrum has two layers. [T] | T |
| Dura mater ends at the level of the second sacral vertebra, becoming the filum terminale. | T |
| Arachnoid mater is the thinnest of all meninges. | T |
| Subarachnoid space is between the dura mater and the arachnoid mater. | F |
| Pia mater is the delicate innermost layer of the meninges. | T |
| 3. Indicate the proper statement(s). |  |
| The central sulcus belongs to the frontal lobe. | F |
| The radial sulcus and diagonal sulcus are located on the parietal lobe. | F |
| The column of fornix and the anterior tubercle of thalamus limit the interventricular foramen. | T |
| The thalamus is the largest concentration of the gray matter of the mesencephalon. | T |
| The anterior wall of the third ventricle belongs to the telencephalon. | T |
| 4. Indicate the proper statement(s) regarding the cavernous sinus. |  |
| Cavernous sinus lies at the base of the greater wing of sphenoid bone. | T |
| Dura mater forms the superior and lateral wall of the cavernous sinus. | T |
| Abducens nerve runs in the lateral wall of the ventricle. | F |
| The nerve that supplies the superior oblique muscle runs through the cavernous sinus. | T |
| The cranial nerve VI runs the cavernous sinus. | T |
| 5. Indicate the proper statement(s). |  |
| The basilar artery arises from the vertebral arteries at the level of the superior border of the pons. | F |
| The vertebral arteries run in the foramen of transverse process of the C1-C7 cervical vertebrae. | F |
| The anterior spinal artery originates from the vertebral artery inferior to the basilar artery. | T |
| The posterior inferior cerebellar artery is the cerebellar branch of the vertebral artery. | T |
| The posterior spinal artery is a branch of the vertebral artery and runs in the posterolateral sulcus. | T |
| 6. Indicate the proper statement(s). |  |
| Central sulcus separates the frontal lobe from the parietal lobe. | T |
| The parahippocampal gyrus is an extension of the cingulate gyrus. | T |
| Precuneus is located between the central sulcus and the parieto-occipital sulcus. | F |
| Front end of cingulate gyrus extends into the subcallosal area. | T |
| Straight gyrus is located in the occipital lobe. | F |
| 7. Indicate proper statements regarding the cerebellum: |  |
| The cerebellum occupies the posterior cranial fossa | T |
| It is attached to the pons by the superior cerebellar peduncle | F |
| It is divided into anterior and posterior lobes by the horizontal fissure | F |
| The cerebellar tonsil is found on its inferior surface | T |
| It derives part of its blood supply from the circles of Willis | F |
| 8. A patient displayed loss of pain, temperature and tactile sensation all on the left side of the body. These symptoms could be caused by a unilateral lesion in which of the following locations: |  |
| Spinal cord | F |
| Medulla | T |
| Pons | T |
| External capsule | F |
| Internal capsule | T |
| 9. Which of the following sensory pathways synapses in the thalamus prior to accessing the cortex? |  |
| Vision | T |
| Hearing | T |
| Fine touch | T |
| Olfaction | F |
| Taste | T |
| 10. Regarding the fourth ventricle: |  |
| It is located in the brainstem. | T |
| It connects to the third ventricle via the cerebral aqueduct. | T |
| It is filled with cerebrospinal fluid. | T |
| It is located anterior to the cerebellum. | T |
| It connects to the central canal of the spinal cord. | T |
| 11. Regarding the basal ganglia: |  |
| They are part of the extra-pyramidal system. | T |
| The head of the caudate nucleus indents the anterior horn of the lateral ventricle. | T |
| The lentiform nucleus comprises the lateral globus pallidus and the medial putamen. | F |
| The claustrum is a thin sheet of white matter between the putamen and insula. | F |
| The lentiform nucleus is bounded medially by the internal capsule. | T |
| 12. The spinal cord is supplied by: |  |
| anterior spinal artery which arises from the basilar artery | F |
| posterior spinal artery which arises from the vertebral artery | T |
| radicular arteries which divide into anterior branch connecting with ventral spinal artery | T |
| anterior spinal artery which receives segmental branches from the ascending cervical artery | T |
| anterior spinal artery which receives segmental branches from the posterior intercostal artery and lateral lumbar arteries | T |
| 13. Cerebral crus contains: |  |
| piramidal fibres | T |
| extrapyramidal fibres | F |
| corticonuclear fibres | T |
| fibres of the anterior spinothalamic tract | F |
| frontopontine fibres | T |
| 14. The interventricular foramen connects: |  |
| the lateral ventricles | F |
| lateral and third ventricle | T |
| third and fourth ventricles | F |
| fourth ventricle and subarachnoid space | F |
| fourth ventricle and central canal of spinal cord | F |
| 15. Which structure(s) belong(s) to the metathalamus: |  |
| tuber cinereum | F |
| mamillary bodies | F |
| lateral geniculate body | T |
| medial geniculate body | T |
| ansa lenticularis | F |
| 16. Which cranial nerves lead the nerve fibers from the ambiguous nucleus? |  |
| Nerve VII | F |
| Nerve IX | T |
| Nerve X | T |
| Nerve XI | T |
| Nerve V | F |
| 17. Indicate proper statements about the cavernous sinus: |  |
| Into cavernous sinus drains superior ophthalmic vein via inferior orbital fissure | F |
| Mandibular nerve is a content of cavernous sinus | F |
| Superior petrosal sinus connects cavernous sinus with internal jugular vein | F |
| Cavernous sinus communicates with pterygoid plexus through the venous plexus of foramen ovale | T |
| Left and right cavernous sinuses are connected by the sphenoparietal sinus | F |
| 18. The cerebellum is an outgrowth of the: |  |
| diencephalon | F |
| mesencephalon | F |
| prosencephalon | F |
| metencephalon | T |
| myelencephalon | F |
| 19. Regarding the fourth ventricle: |  |
| In sagittal section it is triangular with an anterior floor and a roof directed to the apex. | T |
| Upper part of the roof is formed by the superior cerebellar peduncles. | T |
| Cerebrospinal fluid flows cranially from the fourth to the third ventricles. | F |
| Cerebrospinal fluid flows out into the basal cisterns. | T |
| Intraventricular tumors spread through the lateral aperture of the fourth ventricle (foramen of Luschka) into the subarachnoid spaces. | T |
| 20. Indicate the proper statements: |  |
| The lateral sulcus has 3 rami: anterior, ascending and posterior. | T |
| Immediately above the corpus callosum is the cingulate gyrus, which is part of the reticular formation | F |
| The insular lobe on the complete brain is invisible | T |
| The stimulation of the amygdaloid body results in mood changes | T |
| The trochlear nerve leaves the brains on the middle cerebellar peduncle | F |
