# Supplementary material for: Medical students' examinations during the pandemic: Performance and perceptions of anatomy teaching and learning before, during, and after COVID‐19 lockdowns
Source: Anat Sci Educ. 2025 Oct 16;19(1):46–71. doi: 10.1002/ase.70136 (PMC12748042; doi:10.1002/ase.70136)
Supplement: Supplementary file 2 — Table S2. Example list of structures from a final unit practical examination. [file ASE-19-46-s001.docx]

**Example list of structures from a final unit practical examination (central nervous system)**

| 1 | Trunk of corpus callosum |
| --- | --- |
| 2 | Superior temporal gyrus |
| 3 | Olfactory tract |
| 4 | Mamillary body |
| 5 | Optic nerve |
| 6 | Posterior communicating artery |
| 7 | Falx cerebri |
| 8 | Collateral triangle |
| 9 | Pons *(MRI)* |
| 10 | Cerebellar Tonsil *(MRI)* |
| 11 | Thalamus |
| 12 | External capsule |
| 13 | Vertebral artery |
| 14 | Olive |
| 15 | Substantia nigra |
| 16 | Middle cerebellar peduncle |
| 17 | Inferior colliculus |
| 18 | Pulvinar |
| 19 | Basilar artery *(angiography)* |
| 20 | Internal carotid artery *(angiography)* |
